# Patient safety during anesthesia in Ukraine: national audit results

**DOI:** 10.1186/s12871-022-01704-7

**Published:** 2022-05-27

**Authors:** Kateryna Bielka, Iurii Kuchyn, Nataliia Semenko, Uliana Kashchii, Iryna Pliuta

**Affiliations:** Postgraduate Department of Surgery, Anesthesiology and Intensive Care, Bogomolets National Medical University, 13 T. Shevchenko Boulevard, 01601 Kyiv, Ukraine

**Keywords:** Patient safety, Helsinki Declaration, Monitoring in the operating room

## Abstract

**Background:**

Although anesthesiologists are one of the leaders in patient safety, anesthesia in low and low-middle income countries still need improvement in safety mesures with evidence-based practice application. The study aim was to audit the safety principles implementation in the Ukrainian anesthesiologist`s practice.

**Methods:**

The study was held in March 2021-Februrary 2022 by filling out an online questionnarie. The link to the survey was distributed through Ukrainian Anesthesiologists Association (UAA) members emails and also published on UAA webpage and facebook page. The email was sent to 1000 UAA members.

**Results:**

Summary 210 respondents took part in the study. Among the respondents, 79.1% of respondents are aware of the Helsinki Declaration on Patient Safety in Anesthesiology, but only 40,3% declared that the principles of this Declaration had been implemented in their medical institutions. Even though most of the respondents declared that the quality of the work has improved with the application of the Helsinki Declaration, 16% stated, that there is no positive impact.

Most of the medical institutions include mandatory perioperative monitoring, while 17% of hospitals have no access to pulse oximetry for all patients in the operating room and intensive care unit. Concerning using clinical protocols, the one on the treatment of massive bleeding is used in 60.3% of cases, on infection control in 60.5%.

In relation to checklists, 28.2% of respondents have never heard about the WHO Safe Surgery checklist. Checklists for equipment inspection are used in only 27.8% of medical institutions. 72.8% hospitals keep records of anaesthesia complications.

**Conclusion:**

The study showed that significant positive steps are being taken to improve patient safety in Ukraine, where most hospitals comply with the minimum standarts of monitoring during anesthesia. Although there are many challenges for improvement, more hospitals need to implement WHO Safe Surgery and equipment checklists, protocols etc. These areas are a priority for further development in Ukraine.

**Trial registration:**

Clinicaltrials.gov NCT05175976 on 04/01/2022.

**Supplementary Information:**

The online version contains supplementary material available at 10.1186/s12871-022-01704-7.

## Background

Patient safety is critical during the perioperative period. It is due to the significant number of surgical and anesthetic risks during surgery and the postoperative period. The consequences of anesthesia complications have a significant impact on long-term surgical outcomes, patients’ quality of life, morbidity, and mortality [1]. In addition, medical errors have high financial influence, for example, in the United States, they are the eighth leading cause of death, valued at $ 54.6 billion to $ 79 billion, or 6 percent of total annual national health care spending [2].

The Helsinki Declaration on Patient Safety in anesthesiology was introduced in 2010 by the European Society of Anesthesiologists and the European Council of Anesthesiologists [3]. The Declaration is based on many years of research and is a pan-European consensus on the strategy and practical tools needed to maintain patient safety in the perioperative period. Most anesthesiologists associations as well as Ukrainian, have signed a declaration to demonstrate their readiness to use the rules in every hospital and intensive care unit.

Despite the widespread acceptance of high-level principles, there is still some uncertainty about their application and impact in practice [4]. In particular, the 2019 study “Patient safety and the role of the Helsinki Declaration on Patient Safety in Anesthesiology” in 38 European countries [5] showed that despite the generally good level of implementation of the recommendations, there is still a lot of room for improvement in some areas.

The Ukrainian Association of Anesthesiologists has a several educational projects to implement the principles of the Helsinki Declaration. However, the level of compliance with the recommendations can vary significantly depending on the region, the provision of the hospital, and the effectiveness of the institution's internal management. We conducted a survey among anesthesiologists on monitoring standards, checklists availability, and having training and quality control in their departments.

The aim of the study was to evaluate the implementation of the components of the Helsinki Declaration in Ukrainian hospitals, as well as other safety measures during anesthesia.

## Materials and methods

The survey was conducted in March-June 2021 by filling out a standard Google form. The survey design was approved by Bogomolets National Medical University ethical committee (protocol #148 on 7 September 2021). All participants sign the informed consent form before filling the questionnaire. The link to the survey was distributed through Ukrainian Anesthesiologists Association (UAA) members emails and also published on UAA webpage and facebook page. The email was sent to 1000 UAA members. The survey link is https://aaukr.org/bezpeka-patsiyenta-v-operatsijnij/.

To conduct the survey, we formed a questionnaire of 23 items, using the literature that describes in detail the methods of conducting reliable surveys [6, 7]. The questionnaire was designed to ensure sufficient collection of data from respondents on demographics, awareness of the Declaration, and the use in the everyday practice of the measures recommended in it to promote patient safety. However, the structure of the questionnaire allowed people to fill it in a short time. We also provided respondents with more descriptive and detailed answers to some questions if they wished. The questionnaire is in the supplemental material. Multiple options to answer are specificated, when appropriate, other questions were free to answer. The survey was conducted in Ukrainian. A draft version of the questionnaire was piloted with 15 anesthesiologists (native Ukrainian). Only practiticing anesthesiologists, as well as heads of departments, were invited to participate in the survey.

### Statistical analysis

The survey results were imported into a Microsoft Excel spreadsheet for further analysis. Simple descriptive statistics were used. We present categorical data as frequency distribution – numbers and percentages for each value, for visualization we use pie charts. Continious data we present as numbers and visualize as bar graphs. The answers, which included the free text were grouped by topic using simple qualitative techniques.

## Results

In Ukraine currently we have 7500 practicing anesthesiologists. The link to the survey was distributed through email to 1000 UAA members. 210 experienced anesthesiologists took part in the survey, 48.3% of whom perform more than 300 anesthesias per year, 37.1%—from 100 to 300 anesthesias per year. All responders were from hospitals (in-patient). Specialists from most regions of Ukraine were interviewed. The largest share was made by anesthesiologists from Kyiv – 40,8% of respondents, from 20 different medical institutions. Specialists from Dnipro (2,6%), Kharkiv (2,6%), Odesa (3.9%), and Poltava (5,3%) also took part in the survey. The rest of the respondents were evenly distributed among the regions of Ukraine.

**Fig. 1:**
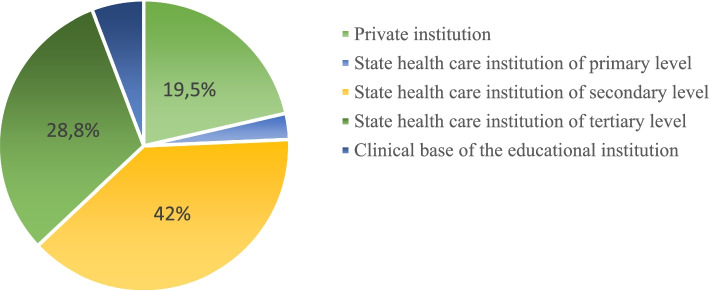
Responder’s workplaces distribution structure

The survey involved anesthesiologists from public hospitals, private hospitals and clinical departments of medical universities (Fig. 1).

According to the results, 79.1% of respondents are aware of the Helsinki Declaration on Patient Safety in Anesthesiology. Among the respondents, only 40,7% stated that the principles of the Helsinki Declaration had been implemented in their medical institutions, and 21.6% about plans to implement them in the near future. At the same time, in 37,7% of medical institutions, the principles of the declaration are not used, and their implementation is not planned.

In 21,9% of medical institutions measures to improve patient safety began to be implemented in 2012–2014, in 2018 – 9,5%, in 2019–2020—16.6% of respondents. Most of the respondents stated that the quality of the department's work has improved and the level of patient safety has increased after the beginning of the application of these principles in their work. At the same time, 16% of respondents have found no positive impact or state that the principles of the declaration complicates their work.

In those medical institutions where the respondents are practicing, the standards of mandatory perioperative monitoring include pulse oximetry (99.5% of cases), blood pressure measurement (95.1%), electrocardiography (85%), capnography (70.9%), temperature monitoring (64.6%), BIS (27.2%), monitoring of central venous pressure – 45.6%, invasive blood pressure—19.9%, gas monitoring—48.1%. Interestingly, some hospitals now provide the use of the latest monitoring systems—TOF, cerebral oximetry, Mdoloris system, ANI monitoring, but, at the same time, 17% of hospitals have no access to pulse oximetry for all patients in the operating room and intensive care unit.

**Fig. 2:**
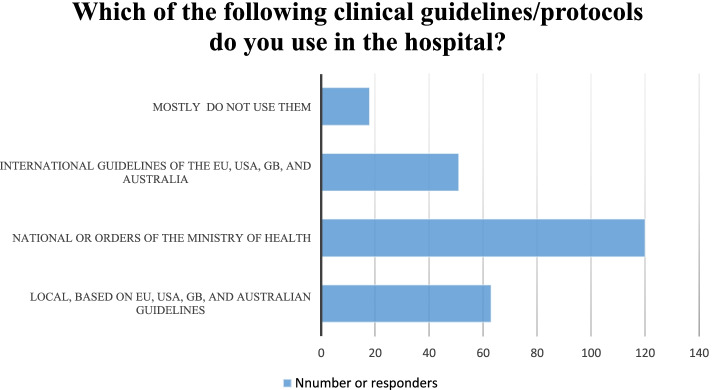
Clinical protocols use in the everyday practice in Ukrainian hospitals

One of the goals of the Helsinki Declaration is all medical institution having the protocols for checking equipment and drugs, management of difficult airways, anaphylaxis, massive bleeding, preoperative preparation, postoperative analgesia, etc. The majority of respondents indicated that they use preoperative examination and preparation protocols (92.4%), postoperative analgesia protocol (83.2%), unfortunately, such important protocols as treatment of massive bleeding (60.3%), difficult airways (72, 2%), infection control (60.5%), are less common. The protocols origin (International, national, local) distribution showed on the Fig. 2. Most doctors indicated that they use the national, local or international protocols, although 10.2% of respondents mostly do not use protocols in their practice. Among the reasons why they are not using protocols, respondents mentioned the lack of interest, time, money, difficulties with learning new technologies for senior colleagues. As we know, anesthesiologists have one of the highest rate of burnout syndrome, with 59,2% being at risk of it and 13,8% meeting it’s criteria [8]. This condition could significantly decrease the quality of medical care, professionalism level and also could be the reason not to use protocols. Othere possible factors are week national health service or local hospital administration regulations.

**Fig. 3:**
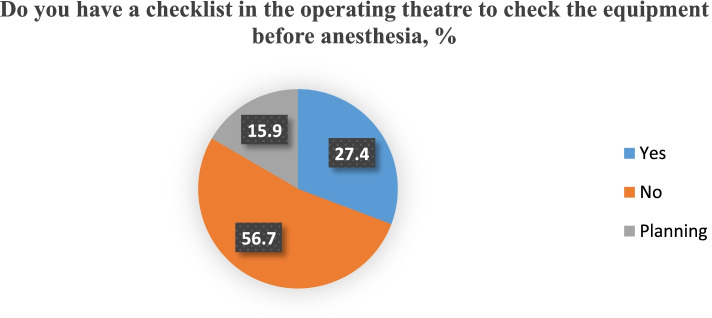
Equipment checklists availability in Ukrainian operating rooms

**Fig. 4:**
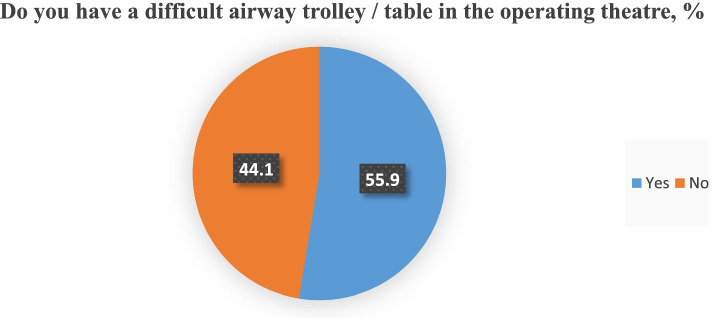
Difficult airway trolleys/tables availability in Ukrainian operating rooms

Unfortunately, the use of the WHO Safe Surgery checklist was very uncommon, with 16.7% of respondents always using such a checklist, and 19.7% are using it sometimes. 28.2% of respondents have never heard of the existence of this document. Checklists for equipment inspection are used in only 27.8% of medical institutions. Difficult airways tables are present in 55.4% (Figs. 3 and 4). 33.7% of hospitals have an emergency notification system.

**Fig. 5:**
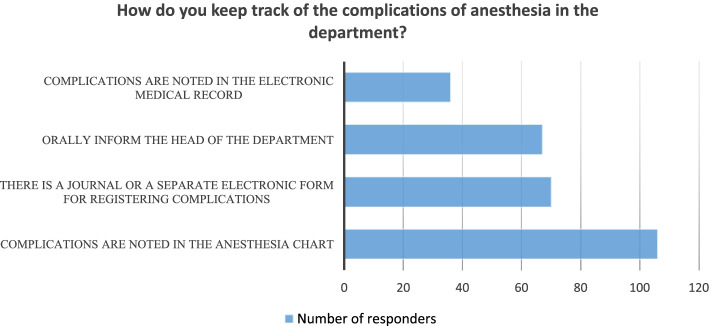
Anesthesia complications reporting in the Ukrainian hospitals

Complication records show that only 72.8% of hospitals keep records of anesthesia complications at all, with only 41% having separate electronic or paper documents to record them. In most institutions, complications are only indicated in the anesthesia card or orally reported to the head of the department (Fig. 5). Also, the majority of physicians-respondents (66.9%) reported the lack of critical incident reporting systems and protocols of critical incidents management.

Intra-clinical examinations of complications or emergencies are always performed in only 34% of medical institutions. Training for doctors on providing emergency care, CPR, airway management 3–5 times a year are held in 7.8% of institutions, mostly such pieces of training are held 1–2 times a year (45.6% of respondents) or not held at all ( 46.6%).

## Discussion

This is the first audit on patient safety in the operating room and Helsinki declaration implementation in Ukraine. The study showed that significant positive steps are being taken to improve patient safety: most hospitals comply with the minimum standarts of monitoring during anesthesia and recovery, providing high prevalence of pulseoxymetry, BP, ECG monitoring etc. Also half of institutions already implement Helsinki declaration principles, others are planning to do it soon. The use of WHO Safety checklist and equipment check documentation before anesthesia is becoming more common. This makes the patients' stay in the operation room and recovery safe and greatly facilitates the work of medical staff.

Despite the overall good results in pulseoxymetry prevalence, there are still 5.2% hospitals where pulseoximetry used in 50–80% of anesthesia cases, this number could even be higher as the study represent respondents from large regional hospitals. In Ukraine we have significant differences between hospitals equipment availability, as we could see from this study many big central hospitals have advanced monitoring technologies (TOF, cerebral oximetry, Mdoloris system, ANI monitoring) and other safety measures (checklists, protocols, emergency notification systems), but, at the same time, we have a lot of small regional hospitals, with little patients load and poor equipment supply (17% of hospitals have no access to pulse oximetry for all patients in the operating room and intensive care unit). The ongoing medical system reform plan to eradicate this hospitals in behalf of bigger ones.

In every second hospital (43%) there is no training among staff on emergency management, cardiopulmonary resuscitation and in 62.9% of cases, there are no clear algorithms for dealing with emergencies in the operating room (including calling for help). The majority of physicians-respondents reported the lack of critical incident reporting systems in their hospitals.

Other authors have analysed Helsinki declaration implementation in European countries, members of European society of anesthesiology (ESA) [5]. Basic monitoring, as recommended by WHO standards, was widely used in hospitals that participated in the survey. The results are close to 100% for pulse oximetry and blood pressure, 98% for ECG, and 96% for capnography. So the main difference was higher prevalence of capnography and ECG monitoring comparing with Ukrainian hospitals.

Authors report that 90% of respondents "always" or "sometimes" use WHO Safe Surgery checklist [5]. In Ukraine, this checklist is "always" or "sometimes" used by 37.4% of respondents. This diffirences could be explained by the lack of National health service orders and documents on safety in anesthesiology, so the hospitals are not directed to improve and reform their safety measures. Other bariers are lack of education as the safety topics was not included in the previous medical university / residency programs. This year we published new residence program in anesthesiology and intensive care, including Safety in anesthesiology block, and also provide postgraduate trainings to improve doctors education and motivation. Other authors report that the most common barrier to checklist implementation was active resistance or passive noncompliance from individuals, most frequently from senior surgeons and/or anesthesiologists [9]. Some studies show that implementation of surgical checklist requires changes in the workflow and increase workload [10].

More than three-quarters of respondents (78.7%) in the European survey [5] said their hospital had an emergency reporting system. In Ukraine, only 45.3% of respondents reported having similar ones in their hospitals. This results also have multiple peasons, at first, educational one (goverment and hospital administration still didn’t pay much attention on safety), and also financial, as hospitals have restricted budget.

So the main implication of our study is finding the priority areas for further patients safety improvement in Ukraine. Among them are further implementation of WHO Safe Surgery and equipment checklists, protocols, critical incidents reporting systems etc.

The limitations of our study is the small sample of respondents and apparently low responsiveness rate, which is not quantifiable according to distribution of the survey via emails and social networks. Also there were no way to verify that respondent was actually an anesthesiologist. About 43% of respondents work in Kyiv, a significant part—in private medical institutions with are much better equipped compared to institutions in other regions of Ukraine.

## Conclusions

In Ukraine, the Helsinki Declaration principles and other safety measures during anesthesia and recovery are being gradually implemented, providing high prevalence of pulseoxymetry, BP, ECG monitoring. Although the use of the WHO "Safe Surgery" checklist is limited. Many departments do not document the equipment check before anesthesia, do not have trolleys for difficult airways, and do not register complications with futher analysis. A small part of hospitals has a critical incident reporting system. These areas are a priority for the further development of patient safety during anesthesia and recovery in Ukraine.

## Supplementary Information

**Additional file 1.**

## Data Availability

The datasets used and/or analysed during the current study available from the corresponding author on reasonable request.

## Abbreviations

ANI: Analgesia nociception index
BIS: Bispectral index
CPR: Cardiopulmonary resuscitation
ECG: Electrocardiography
LA: Local anesthetics
NMU: National medical university
TOF: Train of four
WHO: World Health Organization
