# Supplementary material for: Patient safety during anesthesia in Ukraine: national audit results
Source: BMC Anesthesiol. 2022 May 27;22:164. doi: 10.1186/s12871-022-01704-7 (PMC9137063; doi:10.1186/s12871-022-01704-7)
Supplement: Supplementary file 1 — Additional file 1. [file 12871_2022_1704_MOESM1_ESM.pdf]

Table 1. Questionnaire «Patient safety in the operating room in Ukraine»

1	In which city of Ukraine do you practice or which branch of the Association of Anesthesiologists of Ukraine do you represent ?	Free answer
2.	How many anesthesias do you perform per year?	More than 100<br>100-300<br>More than 300
3.	In which medical institution do you mainly work?	Private institution<br>State health care institution of primary /secondary / tertiary level<br>Clinical base of the educational institution
4.	In which region of Ukraine do you work?	Free answer
5.	Have you heard of the Helsinki Declaration on Patient Safety in Anesthesiology?	Yes<br>No
6.	Are the principles of the Declaration of Helsinki implemented in your hospital?	Yes<br>No<br>Planning
7.	In what year did the components of the	2012-2014

	Declaration of Helsinki start to be implemented in your hospital?	2015-2017<br>2018<br>2019<br>2020<br>Haven't started yet<br>Other answer
8.	How has the implementation of the Declaration of Helsinki affected the work of your hospital/ward?	Free answer
9.	What perioperative monitoring standards are used in your hospital ?	Free answer
10.	What is the prevalence of pulse oximetry in your hospital? What other standards of perioperative monitoring are used in your hospital?	For all the patients in ICU or operation room;<br>For all operation room and some of them in ICU<br>For 70-80% in ICU or operation room;
11.	Which of the following clinical guidelines/protocols/routes do you use in the hospital?	Postoperative anesthesia<br>Infection control<br>Massive bleeding<br>Local anesthetic

		intoxication<br>Anaphylaxis<br>Difficult airways<br>Marking of syringes<br>Equipment and medicines check<br>Preoperative examination and preparation
12.	What are the most common protocols used in your hospital?	Local, based on International guidelines<br>National protocols<br>International guidelines of the EU, USA, GB and Australia<br>Mostly we do not use
13.	If you mostly do not use protocols, state the probable reason?	Free answer
14.	Do you use the WHO Safe Surgery checklist or its local analogs?	Always<br>Sometimes<br>Don`t use

		Don`t have the information about this checklist
15.	Do you report mortality in your ward/hospital?	Yes<br>No<br>Don`t know
16.	Do you keep records of anesthesia complications in your department?	Yes<br>No<br>Don`t know
17.	If so, how do you keep track of the complications of anesthesia in the ward?	Complications are noted in the anesthesia chart<br>There is a journal or a separate electronic form<br>Orally inform the head of department<br>Complications are noted in the electronic medical record
18.	18. Do you keep records of emergencies in the operating room (Intraoperative myocardial	Yes, have separate document/ journal for

	ischemia, arrhythmia, heavy bleeding, bronchospasm, aspiration, hemolytic reaction, LA intoxication, etc.)?	registration<br>Yes, orally inform the head of department<br>No
19.	Do you have an operating table for difficult airways?	Yes<br>No
20.	Do you have in the operation room a checklist to check the equipment before anesthesia?	Yes<br>No<br>Planning
21.	Does your hospital have approved checklists or emergency algorithms in the operating room?	Yes<br>No
22.	Does your hospital have an emergency notification system (eg, circulatory arrest) or an algorithm to report, and in what order?	Yes<br>No
23.	Does your hospital provide training or education for emergency physicians / CPR / airway management, etc.?	Yes, 3-5 times a year<br>Yes, 1-2 times a year<br>No
